# Metagenomic comparison of the faecal and environmental resistome on Irish commercial pig farms with and without zinc oxide and antimicrobial usage

**DOI:** 10.1186/s42523-023-00283-3

**Published:** 2023-12-11

**Authors:** Daniel Ekhlas, José F. Cobo Díaz, Raúl Cabrera-Rubio, Elena Alexa, Juan M. Ortiz Sanjuán, Edgar Garcia Manzanilla, Fiona Crispie, Paul D. Cotter, Finola C. Leonard, Héctor Argüello, Catherine M. Burgess

**Affiliations:** 1 Department of Food Safety, Teagasc Food Research Centre, Ashtown, Dublin, Ireland; 2 School of Veterinary Medicine, University College Dublin, Dublin, Ireland; 3 Pig Development Department, Teagasc Moorepark, Fermoy, Co. Cork, Ireland; 4 Department of Food Hygiene and Technology, Universidad de León, León, Spain; 5 Teagasc Food Research Centre, Moorepark, Fermoy, County Cork, Ireland; 6 APC Microbiome Institute, University College Cork, Cork, County Cork, Ireland; 7 Grupo de Genómica y Mejora Animal, Departamento de Genética, Facultad de Veterinaria, Universidad de Córdoba, Córdoba, Spain; 8 VistaMilk SFI Research Centre, Fermoy, Co. Cork, Ireland; 9 Animal Health Department, Veterinary Faculty, Universidad de León, León, Spain

**Keywords:** Antimicrobial resistance, Swine, Heavy metals, Zinc oxide, Metagenomics, Resistome

## Abstract

**Background:**

Antimicrobials and heavy metals such as zinc oxide (ZnO) have been commonly used on Irish commercial pig farms for a 2-week period post-weaning to help prevent infection. In 2022, the prophylactic use of antimicrobials and ZnO was banned within the European Union due to concerns associated with the emergence of antimicrobial resistance (AMR) and contamination of the environment with heavy metals. In this study, faecal and environmental samples were taken from piglets during the weaning period from ten commercial farms, of which five farms used antimicrobial or ZnO prophylaxis (AB-ZnO farms) and five which had not used antimicrobials or ZnO for the previous 3 years (AB-ZnO free farms). A total of 50 samples were compared using a metagenomic approach.

**Results:**

The results of this study showed some significant differences between AB-ZnO and AB-ZnO free farms and suggested positive selection for AMR under antimicrobial and ZnO treatment. Moreover, strong differences between environmental and faecal samples on farms were observed, suggesting that the microbiome and its associated mobile genetic elements may play a key role in the composition of the resistome. Additionally, the age of piglets affected the resistome composition, potentially associated with changes in the microbiome post-weaning.

**Conclusions:**

Overall, our study showed few differences in the resistome of the pig and its environment when comparing AB-ZnO farms with AB-ZnO free farms. These results suggest that although 3 years of removal of in-feed antimicrobial and ZnO may allow a reduction of AMR prevalence on AB-ZnO farms, more time, repeated sampling and a greater understanding of factors impacting AMR prevalence will be required to ensure significant and persistent change in on-farm AMR.

**Supplementary Information:**

The online version contains supplementary material available at 10.1186/s42523-023-00283-3.

## Introduction

Antimicrobial resistance (AMR) is a global burden and a threat for human, animal, and environmental health, with an estimate of 4.95 million deaths in 2019 alone due to bacterial AMR [1]. Livestock production is a significant contributor to antimicrobial use (AMU), through animal treatment and prophylactic use [2]. Heavy metals such as zinc oxide (ZnO) are used in pig production for prevention of disease and for their growth and performance enhancement effects on pigs, when supplemented at therapeutic concentrations (2500–3000 ppm) for 2 weeks post-weaning [3]. Moreover, ZnO is also used for treatment of post-weaning diarrhoea. Thus, since the EU began to ban the non-essential use of antimicrobials as growth-promoters in 1999 (coming into full effect in 2006) to tackle AMR development in livestock production, ZnO has served as an alternative on farms for these antimicrobials [4]. However, due to the threat posed by AMR and its association with AMU and heavy metal usage, as well as environmental concerns, prophylactic use of antimicrobials and ZnO was banned in January (Regulation (EU) 2019/6 on Veterinary Medicinal Products) and June 2022 (Regulation (EU) 2019/4 on Medicated Feed; Committee for Veterinary Medicinal Products in the framework of Article 35 of Directive 2001/82/EC) respectively [5, 6].

In the past decade, several studies investigated the effects of in-feed antimicrobials on the porcine microbiome and resistome. Metagenomics-based observational studies such as those by Munk et al. [7] or Van Gompel et al. [8], which examined associations between AMU and AMR on pig farms in nine European countries, reported clear evidence of AMU–AMR associations. However, far less is known about associations between on farm ZnO usage and AMR. The majority of studies investigating associations between ZnO and AMR were conducted under controlled conditions with a small sampling population and may not fully reflect the complexity of commercial farm environments. Based on the studies that have taken place, ZnO usage has been associated with the presence of genes conferring resistance to aminoglycosides, MLSP (macrolides, lincosamides, streptogramins, and pleuromutilins), beta-lactams, polymyxin, and herbicides [9–11]. Factors such as internal biosecurity protocols, AMU, mobile genetic elements (MGEs), and the persistence of AMR bacteria in the environment may also have significant effects on on-farm resistomes [8, 12]. Therefore, it is crucial to consider these factors in studies to gain a better understanding of the effects of AMU and ZnO on the development and spread of AMR on pig farms.

The application of next-generation sequencing methods such as shotgun metagenomics to investigate AMR has become increasingly popular in recent years. Metagenomics facilitate multiple insights when studying the association of antimicrobial resistance genes (ARGs) with specific taxa, MGEs, lateral gene transfer (LGT) events, and microbial functions. Furthermore, metagenomics is not limited to culturable species and does not require species isolation, cultivation, or enrichment, which can be time- and cost-consuming [13].

However, challenges remain when carrying out such metagenomics-based investigations. These include, for instance, (1) low sample sizes due to high sequencing costs, which can significantly affect statistical observations made and representativeness and coverage of target population; (2) study design—randomized trial versus cross-sectional study, which both have their advantages and limitations in making clear associations between experimental conditions and observations; (3) transportation and storage, which can affect the microbial composition of the samples collected prior to sequencing; and (4) contamination and/or lack of controls, which makes it difficult to distinguish microbial contaminants originating from the utensils used for sample preparation, storage, and DNA extraction from the microbial composition in the actual sample. Moreover, in-depth understanding and the involvement of trained personnel is often necessary to allow accurate computational analysis of sequences [14].

In this observational study, a metagenomics approach was used to examine the effects of AMU and ZnO prophylactic treatment for 2 weeks post-weaning on the porcine resistome by taking faecal and environmental samples from five farms that were all using ZnO and either sulphadiazine-trimethoprim or amoxicillin trihydrate and five farms that had not used ZnO or antimicrobials for prophylactic purposes for the previous 3 years. It is important to note that in this study, antimicrobial and ZnO was considered as one treatment group to reflect common practice on many Irish pig farms. The aim of this study was to investigate the effects of antimicrobial and ZnO removal from commercial farm practices on AMR prevalence and persistence.

## Results

### Abundance of antimicrobial, biocide, and heavy metal resistance genes

**Fig. 1:**
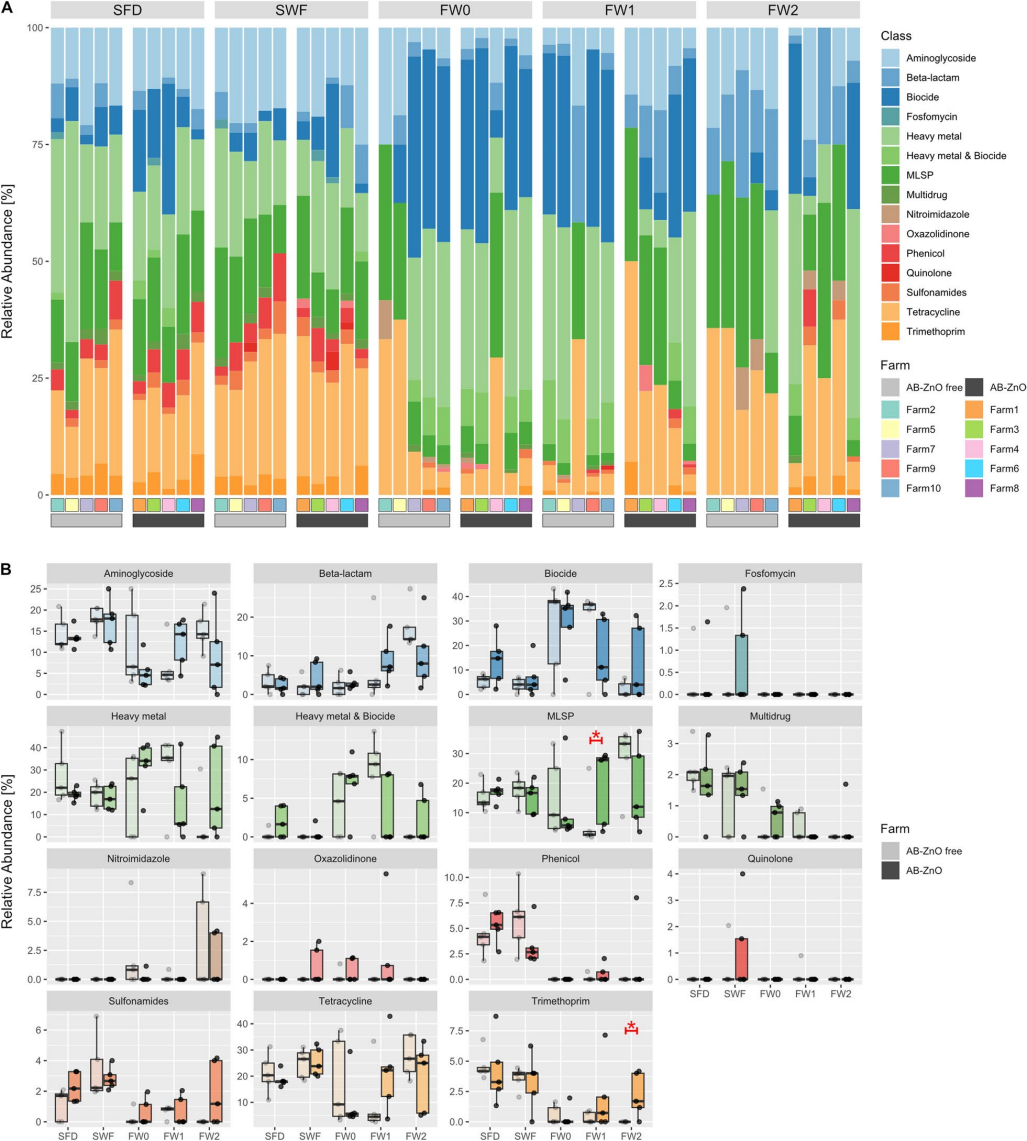
Relative abundance of antimicrobial, heavy metal, and biocide resistance classes, with **A** showing the resistome composition of each sample on each farm and **B** comparison of treatment types [AB-ZnO (darker colours; indicated by dark grey in legend) and AB-ZnO free farms (lighter colours; indicated by light grey in legend)] per sample. Treatment types were compared per sample type using the Mann–Whitney U test with α = 0.05. Significant differences are indicated in red by an asterisk (*p* < 0.05). SFD, swab samples of cleaned feeders and drinkers of empty cleaned pens; SWF, swab sample of walls and floor of the pens prior to introduction of weaned piglets; FW0, faecal samples at the day of weaning; FW1, faecal samples 1 week post-weaning; FW2, faecal samples 2 weeks post-weaning

In this observational study, a metagenomics approach was used to compare five AB-ZnO free farms with five farms that used ZnO and antimicrobials post-weaning (AB-ZnO farms). Samples included environmental swab samples of (i) cleaned drinkers and feeders and (ii) cleaned walls and floors, both in empty pens. Faecal samples were collected (iii) at day of weaning (approximately 28 days of age), (iv) 1 week post-weaning and (v) samples 2 weeks post-weaning. The most abundant ARGs in environmental samples, independently of treatment, were those associated with resistance to tetracycline (mean (${\bar{\text{x}}}$) = 22.52%), MLSP (${\bar{\text{x}}}$ = 16.28%), and aminoglycoside (${\bar{\text{x}}}$ = 15.73%). Together with metal resistance genes (MRGs) (${\bar{\text{x}}}$ = 20.73%) and biocide resistance genes (BRGs) (${\bar{\text{x}}}$ = 7.43%), these resistance genes accounted for more than 80% of the environmental farm resistome. Similar observations were made for faecal samples in which the predominant ARGs were those associated with resistance to tetracycline (${\bar{\text{x}}}$ = 17.56%), MLSP (${\bar{\text{x}}}$ = 16.62%), and aminoglycoside (${\bar{\text{x}}}$ = 10.02%). However, the abundance of BRGs was, in particular, much higher in faecal samples (${\bar{\text{x}}}$ = 19.31%) compared to environmental samples (${\bar{\text{x}}}$ = 7.43%), while MRGs showed a similar abundance to BRGs (environmental: ${\bar{\text{x}}}$ = 20.73%; faecal: ${\bar{\text{x}}}$ = 20.53%) across both categories of sample type (Fig. 1A). Genes associated with resistance to fosfomycin were only observed in environmental samples, while genes linked with resistance to nitroimidazole were only observed in faecal samples (Fig. 1).

Statistical comparisons via the Mann–Whitney U test revealed significant differences between AB-ZnO farms and AB-ZnO free farms in that MLSP and trimethoprim resistance determinants were more abundant in faecal samples from AB-ZnO farms, relative to faecal samples from the AB-ZnO free farms, 1 week post-weaning (*p* = 0.0317) and for 2 weeks post-weaning (*p* = 0.0254), respectively (Fig. 1B). Interestingly, increases of sulfonamide and trimethoprim resistance on AB-ZnO farms were observed post-weaning, which were not observed for the AB-ZnO free farms. Moreover, phenicol and multidrug resistance (genes encoding resistance to a number of antimicrobial classes) was more abundant in environmental than faecal samples.

**Fig. 2:**
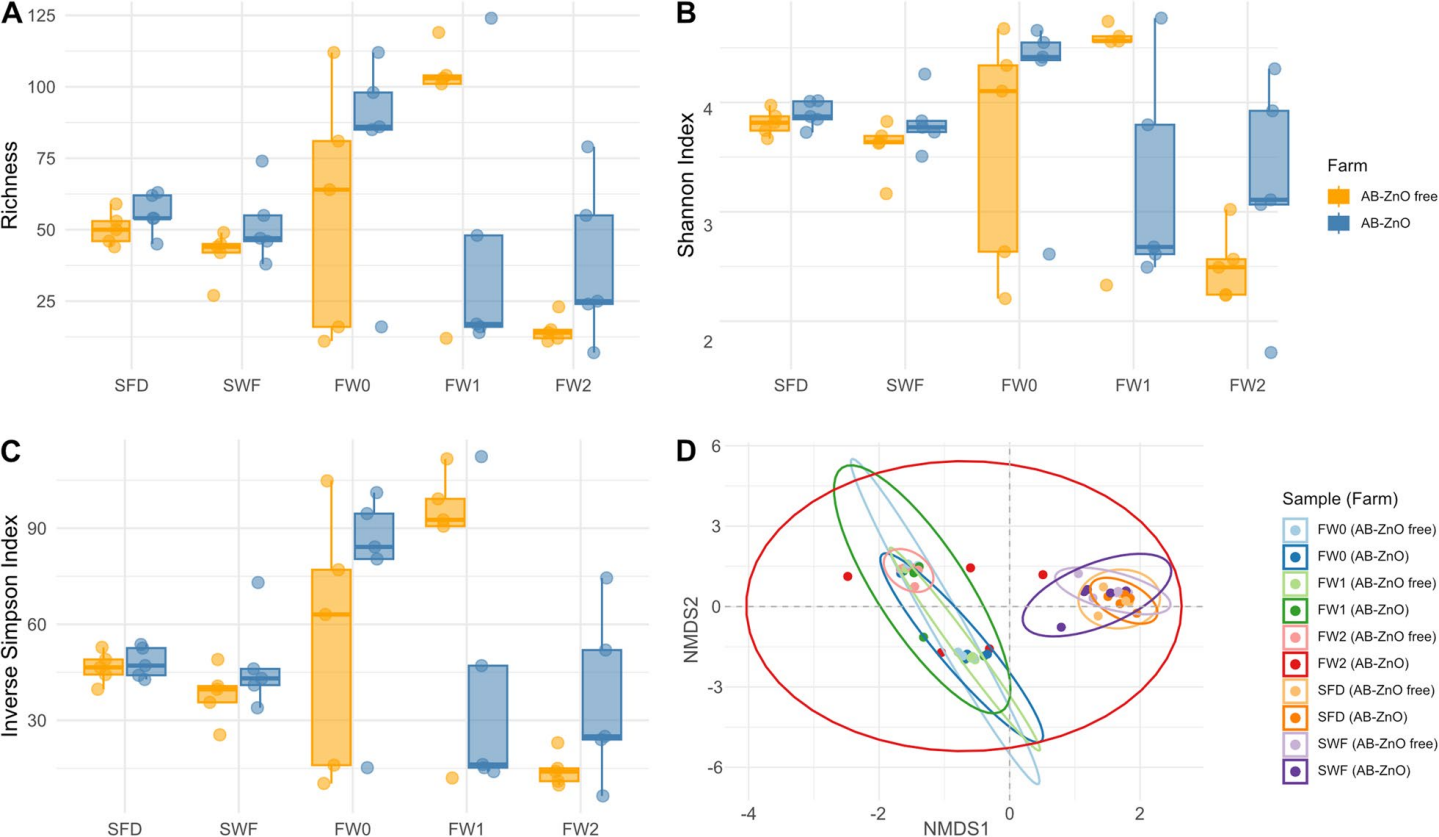
Alpha and beta diversity indices of antimicrobial, biocide, and metal resistance of farm samples. **A**–**C** Richness, Shannon, and Inverse Simpson indices of farm samples. **D** Non-metric dimensional scaling (NMDS) plots based on Bray–Curtis dissimilarity of ARGs, BRGs, and MRGs. SFD, swab samples of cleaned feeders and drinkers of empty cleaned pens; SWF, swab sample of walls and floor of the pens prior to the introduction of weaned piglets; FW0, faecal samples at the day of weaning; FW1, faecal samples 1 week post-weaning; FW2, faecal samples 2 weeks post-weaning

No significant differences between treatment types were observed for alpha diversity indices and richness of resistance genes in environmental and faecal samples (Fig. 2A–C). Here, richness was defined by the number of resistance genes found in each sample, alpha diversity indices such as the Shannon index and the inverse Simpson index were defined as a measure of richness and evenness of resistance genes. Thus, while the Shannon index is weighted based on the overall proportion of resistance genes per sample, the inverse Simpson index was more strongly affected by more abundant resistance genes compared to less abundant ones in our study [15, 16].

It was noted that, for environmental samples, richness and alpha diversity indices were numerically, but not significantly, higher on AB-ZnO farms. Similar observations were made at the day of weaning (FW0) and 2 weeks post-weaning (FW2). However, 1 week post-weaning (FW1), faecal samples showed non-significantly higher values on AB-ZnO free farms compared to AB-ZnO farms. Comparably, beta diversity analysis based on Bray–Curtis dissimilarities using non-metric multidimensional scaling (NMDS) also showed no significant differences between treatment types in accordance with the results of the pairwise PERMANOVA analysis (Fig. 2D). Interestingly, environmental samples of both treatment types formed a distinct cluster with small dispersion in the NMDS plot. These strong similarities between environmental samples were also seen in the alpha diversity analysis. For faecal samples, a stronger dispersion was observed compared to environmental samples. The results of the pairwise PERMANOVA analysis confirmed significant differences (*p* < 0.05) between faecal and environmental samples, independent of treatment type. For FW2 however, dispersion of samples from AB-ZnO farms compared to AB-ZnO free farms was non-significantly higher. These differences were also reflected in the alpha diversity analysis.

### Mobilome and its associated resistance genes

**Fig. 3:**
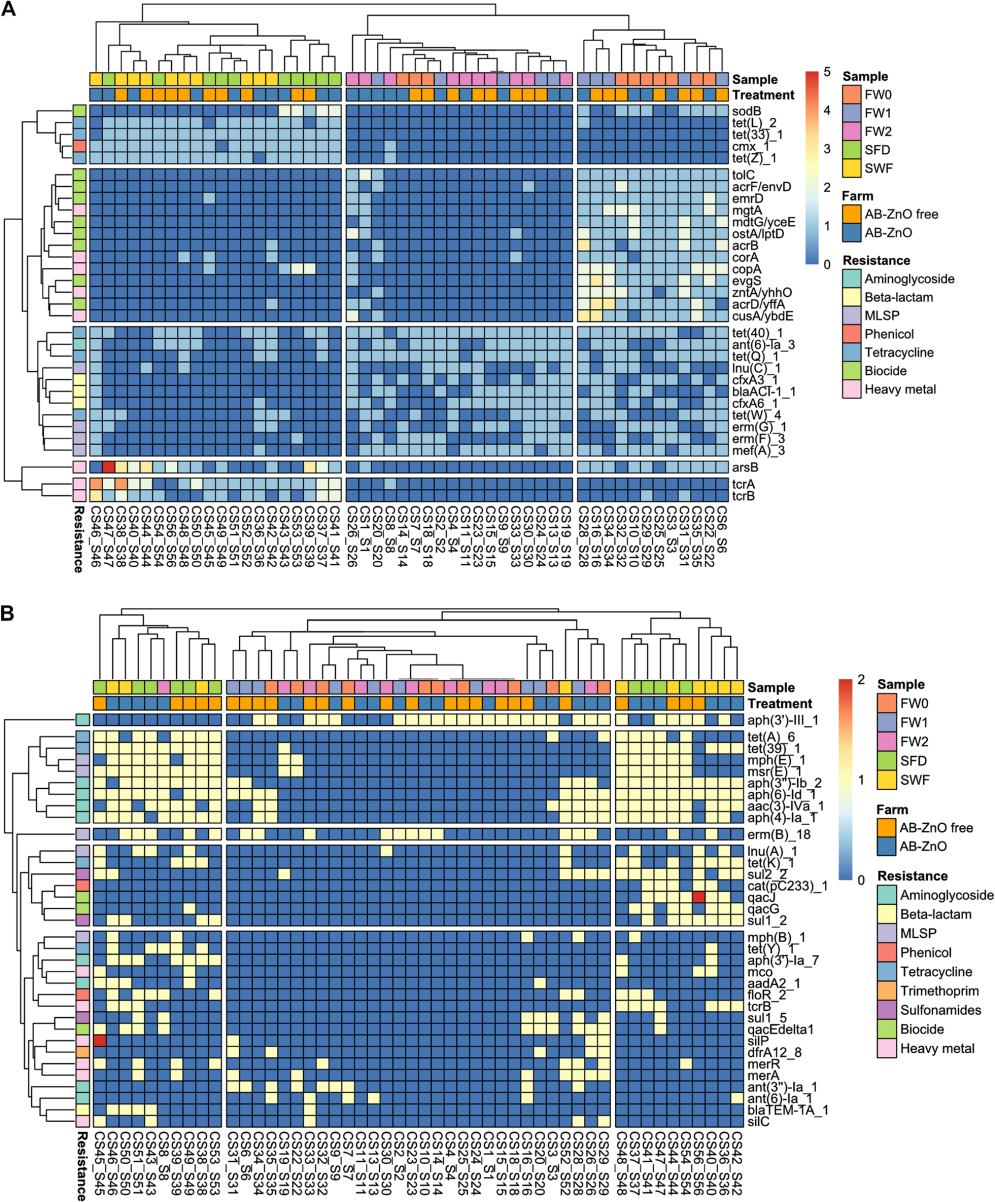
Heatmap of the most abundant resistance genes on chromosomal and plasmidic contigs. Most abundant resistance genes are based on number of hits, found on **A** chromosomal (threshold > 15 total hits based on 30 genes with highest number of hits) and **B** plasmidic contigs (threshold > 4 total hits based on 30 genes with highest number of hits). Dendrograms clusters are based on the Euclidean distance. SFD, swab samples of cleaned feeders and drinkers of empty cleaned pens; SWF, swab sample of walls and floor of the pens prior to introduction of weaned piglets; FW0, faecal samples at the day of weaning; FW1, faecal samples 1 week post-weaning; FW2, faecal samples 2 weeks post-weaning

After separating chromosomal and plasmidic contigs using the software Platon, the most abundant resistance genes were plotted on two different heatmaps (Fig. 3). In the chromosomal-based heatmap, environmental samples (environmental cluster), faecal samples at the day of weaning (weaning cluster), and faecal samples 1 and 2 weeks post-weaning (post-weaning cluster) formed three distinct clusters based on Euclidean distance. In the plasmidic-based heatmap, separation based on Euclidean distance was only observed for faecal and environmental samples, forming one faecal and two environmental clusters. Similar to the previous observations, AB-ZnO and AB-ZnO free farms did not differ greatly. The predominant resistance genes on plasmidic contigs were much more diverse compared to chromosomal contigs. Based on chromosomal contigs, the tetracycline resistance genes *tet*(L), *tet*(33), and *tet*(Z); the phenicol resistance gene *cmx*; and the copper resistance genes *tcrA* and *tcrB* were mainly found in environmental samples. In addition to these genes, the arsenic resistance gene *arsB* and the hydrogen peroxide (biocide) resistance gene *sodB* were found in samples of the environmental and weaning cluster. Moreover, BRGs and MRGs were observed with the highest prevalence in the weaning clusters. The majority of the most prevalent ARGs (11 of 15 ARGs) on chromosomal contigs were found in faecal samples, including genes encoding resistance to tetracycline, MLSP, beta-lactam, and aminoglycoside.

On plasmidic contigs, only three biocide resistance genes, namely *qacJ*, *qacG*, and *qacEΔ1* were observed as part of the environmental clusters. The predominant ARGs in the environmental clusters were the tetracycline resistance genes *tet*(A), *tet*(39); the aminoglycoside resistance genes *aph(3'')-Ib*, *aph(6)-Id*, *aph(4)-Ia*, and *aac(3)-IVa*; and the MLSP resistance genes *mph*(E) and *msr*(E).

**Fig. 4:**
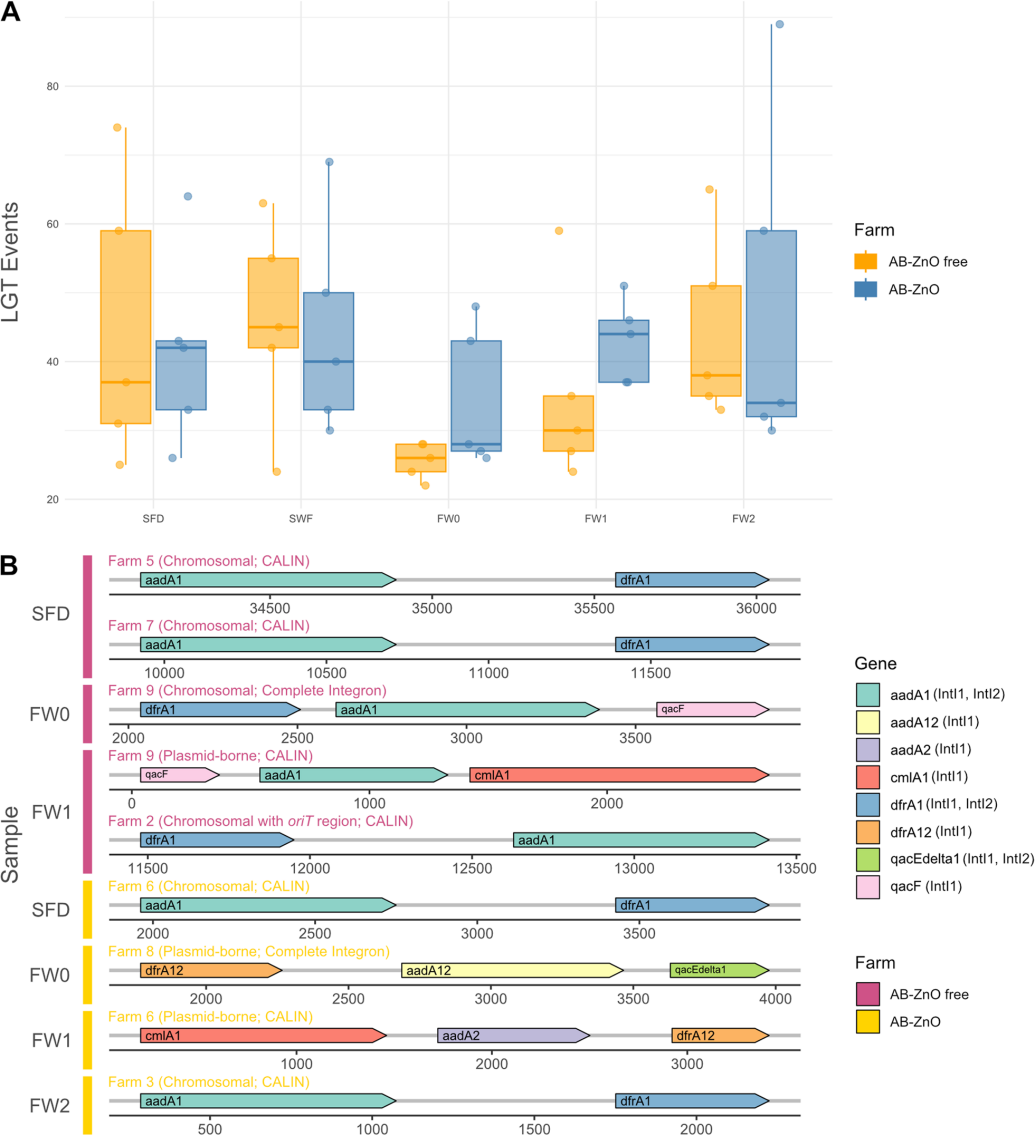
Lateral gene transfer events and integrons in farm samples. Lateral gene transfer events (**A**) and integrons with at least two ARGs, MRGs, or BRGs found in all samples (**B**) are shown. Potential integron classes are written in brackets for each gene in accordance with entries in the INTEGRALL database. SFD, swab samples of cleaned feeders and drinkers of empty cleaned pens; SWF, swab sample of walls and floor of the pens prior to introduction of weaned piglets; FW0, faecal samples at the day of weaning; FW1, faecal samples 1 week post-weaning; FW2, faecal samples 2 weeks post-weaning

To investigate the occurrence of LGT events and the spread of antimicrobial resistance, the WAAFLE software tool was used. No statistical differences between treatment types were observed for the occurrence of LGT events, although their occurrence was numerically higher on AB-ZnO farms for piglets at the day of weaning (FW0) and 1 week post-weaning (FW1). At 2 weeks post-weaning, the occurrence of LGT events was similar again for both treatment types (Fig. 4A). ARGs were transferred in only two LGT events, namely the *aadD_1* gene in both cases, encoding for an aminoglycoside adenyltransferase. Both LGT events were identified in swab samples of walls and floor on different AB-ZnO farms.

Integrons on plasmids and chromosomes were identified using IntegronFinder 2.0. In total, 157 integron elements [118 CALINs (lacks integron-integrases), 10 complete integrons, 29 In0 (lacks gene cassette)] were identified. The majority of integrons was found on AB-ZnO free farms (n = 99), most of them reported as CALINS (n = 75). Moreover, integrons were predominantly found in environmental samples, with 77 integrons on AB-ZnO free farms (77.78% of total integrons) and 40 integrons on AB-ZnO farms (68.97% of total integrons). Only 35 of all 157 identified integrons carried ARGs. Nine of these 35 integrons carried multiple resistance genes and are visualized in Fig. 4B. The resistance genes *aadA1* (confers aminoglycoside resistance) and *dfrA1* (confers trimethoprim resistance) were the most frequently occurring genes on ARG-harbouring integrons, followed by *qacF* (confers resistance to quaternary ammonium compounds) and *dfrA12*. The chloramphenicol resistance gene *cmlA1* and the aminoglycoside resistance gene *ant(3'')-Ia* were additionally found on some integrons. The majority of these ARGs and BRGs were previously reported as being encoded on class 1 integrons but some were associated with IntI2, according to the INTEGRALL database (http://integrall.bio.ua.pt/).

### Metagenome-assembled genomes and their associated resistance genes

To further investigate the association of ARGs, BRGs, and MRGs with specific taxa, metagenome-assembled genomes (MAGs) were generated and analysed for the presence of resistance genes. In total 486 MAGs were generated from the 50 samples, of which 197 MAGs belonged to a unique species, 43 unique species carried ARGs, and 19 unique species carried BRGs and MRGs. Additional information regarding sample and farm origin of MAGs can be extracted from Additional file 1.

**Fig. 5:**
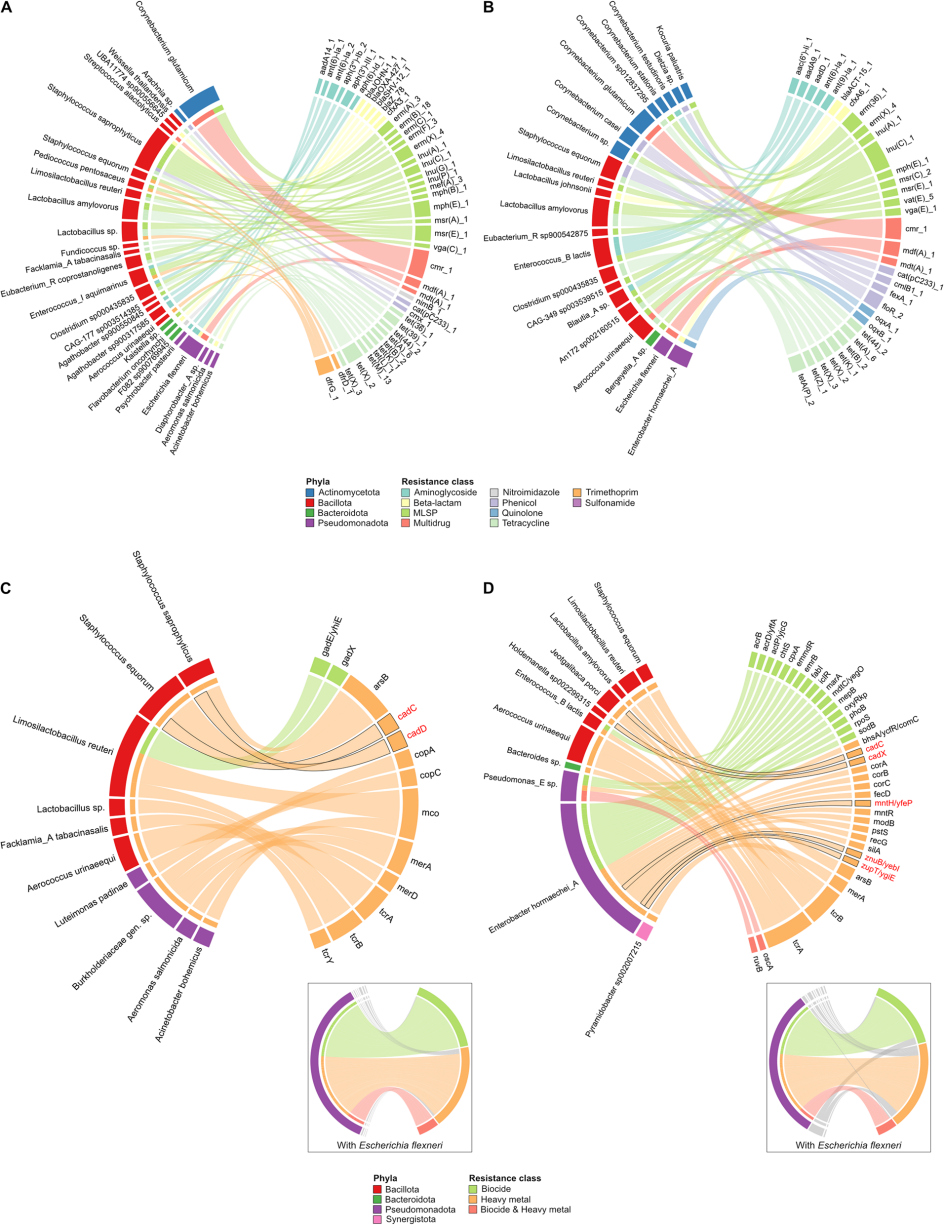
Resistance genes and their associated MAGs. Occurrence of **A**, **B** antimicrobial resistance and **C**, **D** heavy metal and biocide resistance genes in assembled MAGs (taxonomic level: species) in samples of AB-ZnO free (**A**, **C**) and AB-ZnO farms (**B**, **D**). Due to the high prevalence of MRGs and BRGs in *E. flexneri* strains, this strain is excluded in figure parts **C** and **D**. The original graph including E. flexneri is additionally added on the bottom right of each figure part, highlighting only resistance genes found in *E. flexneri*. MRGs conferring resistance to zinc are written in red font

The comparison of MAG-encoded AMR between AB-ZnO and AB-ZnO free farms revealed that the majority of ARGs was associated with species of the phylum Bacillota (Fig. 5A, C). The diversity of species and ARGs on AB-ZnO free farms was higher compared to AB-ZnO farms. Furthermore, Pseudomonadota was the second most diverse phylum on AB-ZnO free farms, followed by Bacteroidota and Actinomycetota, while on AB-ZnO farms, Actinomycetota was the second most diverse phylum, followed by Pseudomonadota.

Conversely, a comparison of treatment types based on the presence of BRGs and MRGs showed opposing results (Fig. 5B, D). While the main carrier of all these resistance genes was identified as *Escherichia flexneri*, the overall diversity of phyla and resistance genes was higher on AB-ZnO farms.

Since *E. flexneri* carried the majority of BRGs and MRGs, possibly due to a bias of the database towards lab strains such as *E. coli*, *E. flexneri* was excluded from further analysis to facilitate the observation of differences between treatment types. It was observed that only two genes, namely *cadC* and *cadD*, predicted to confer resistance to zinc on AB-ZnO free farms, were associated with the species *Staphylococcus equorum* and *S. saprophyticus*, respectively. On AB-ZnO farms, five different genes conferring resistance to zinc were identified, of which *mntH/yfeP, znuB/yebI,* and *zupT/ygiE* were associated with *Enterobacter hormaechei.* This species was also associated with the quinolone resistance genes *oqxA* and *oqxB*. The other two resistance genes *cadC* and *cadX* were associated with *Jeotgalibaca porci* and *Holdemanella* sp002299315.

## Discussion

This observational metagenomics-based farm study is one of the first to describe the effects of combined prophylaxis of ZnO and antimicrobials post-weaning in pigs. Furthermore, its inclusion of total, chromosomal, and plasmidic contig analysis together with analysis of MAGs is notable. The various approaches taken have respective advantages and disadvantages. For instance, analysis of total contigs may provide an overview of the resistome on farms, but does not allow one to identify which species or MGEs play a key role in the development and spread of AMR. Using plasmid identification tools such as Platon allows the distinction between chromosomal and plasmidic elements. However correct binning of plasmids remains challenging due to small sequence fragments and high plasmid numbers [17, 18]. Nevertheless, plasmid binning can give greater insights relating to the association of MGEs with the resistome. Similarly, metagenomic binning, i.e., assembly of MAGs, has a great advantage in identifying microbial carriers of resistance genes, but can result in a great loss of underrepresented microbial species and genes, such as virulence genes, carried on short contigs after assembly [19]. Consequently, comparing and analysing on multiple assembly levels may provide greater insights into the resistome by overcoming the unique limitations of each approach. Further developments in bioinformatics and sequencing methods will likely allow even greater insights in the future.

The first analysis of contig-based resistomes showed that resistance to tetracycline, MLSP, aminoglycoside, and beta-lactam were common among all samples, independent of sample or treatment type (contributed to 69.57–100% of the resistome in samples). These results are in accordance with previous findings of Mencía-Ares et al. [20], Joyce et al. [21], and Holman Devin et al. [22] in studies undertaken in Spain, Ireland and Canada respectively. This contig-based comparison of resistomes on AB-ZnO and AB-ZnO free farms revealed few differences, with only two of 75 pairwise comparisons per sample type and resistance class showing significant differences, including MLSP resistance being significantly higher in faecal samples 1 week post-weaning on AB-ZnO farms and trimethoprim resistance being significantly higher in faecal samples 2 weeks post-weaning on AB-ZnO farms. Moreover, increases of sulfonamide resistance were additionally observed on AB-ZnO farms post-weaning, although the differences were not significant.

A study by Wang et al. [12] observed that the use of sulfonamides was associated with increased resistance to sulfamethoxazole-trimethoprim. Similarly, a study of commensal *E. coli* conducted by Mazurek et al. [23] reported an association between trimethoprim and sulphamethoxazole metaphylaxis and increased resistance to trimethoprim. Moreover, Yue et al. [24] reported an association of the use of sulfonamides with the MLSP resistance gene *erm*(X) in their study. Separately, associations between ZnO usage at therapeutic (2500 ppm) and dietary (40–110 ppm) concentrations with the MLSP resistance genes *lnu*(C) and *erm*(G) were also reported by Pieper, et al. [9]. Regarding the other three AB-ZnO farms in our study that used amoxicillin in combination with ZnO post-weaning, a study of Zeineldin, et al. [25], who investigated the effects of perinatal single dose treatment of piglets with procaine penicillin G among others, reported associations of procaine penicillin G treatment with MLSP resistance gene *erm*(B) and the tetracycline resistance gene *tet*(W). Therefore, penicillin-like antimicrobials such as amoxicillin may also be associated with MLSP resistance.

Interestingly, no associations between amoxicillin prophylaxis with resistance to beta-lactams was observed in the current study, in fact on the contrary. Reasons for this could be associations of beta-lactam resistance genes such as *cfxA6* with strains such as *Prevotella* sp. that increase in abundance post-weaning and are required for the digestion of solid feed [22]. This could also explain the lack of a significant increase in beta-lactam resistance at 2 weeks post-weaning on AB-ZnO free farms. Nevertheless, it is likely that different antimicrobials affect the on-farm resistome differently. However, in the current study no distinction between AB-ZnO farms could be made, suggesting that future observational studies should focus on investigating a larger number of farms with similar treatments from the same antimicrobial class.

Comparable observations were made in a previous study of ours where we compared the same farms based on analysis of AMR *E. coli*. Although no difference in total *E. coli* numbers between treatment types was observed, resistance to apramycin, trimethoprim, tetracycline, streptomycin, chloramphenicol, and multidrug resistance was more frequently observed on AB-ZnO farms [26]. In the current metagenomics study these differences between treatment types were less visible, which might be due to the low sample number, but also due to the greater complexity of resistomes as part of the microbial community. Moreover, the considerable variation in the resistance profiles of faecal samples (Figs. 1B, 2) is likely to mean that the number of samples collected did not provide a fully representative picture of the two farm treatment types. For instance, as seen in Fig. 1B, MLSP resistance at 1 week post-weaning (FW1) was only significantly different between treatment types, due to three AB-ZnO farms showing higher resistance than the five AB-ZnO free farms, while the other two AB-ZnO farms showed similar values to the AB-ZnO free farms in terms of relative abundance. Thus, the results of the study should be interpreted with this caveat in mind.

When comparing sample types, environmental and faecal samples differed greatly from each other. For instance, some ARGs conferring resistance to fosfomycin were only found in environmental samples, while resistance to nitroimidazole was only observed in faecal samples. Furthermore, biocide resistance was more abundant in faecal samples. However, differences in alpha diversity indices or richness of resistance genes were not observed. This may be due to potential associations of the microbiome and mobilome with the resistome as suggested by Munk et al. [7] and Muurinen et al. [10]. Thus, although pig faeces are a frequent contaminant of the environment, microbiomes may differ, especially after cleaning of the environment. Persisting microbes in the cleaned environment may be carried over between pig batches and thereby allow the transmission and persistence of AMR on farms [27]. Interestingly, richness and alpha diversity indices of the resistome decreased with age of the piglets on AB-ZnO free farms. This decrease was less strong for AB-ZnO farms. Similar findings were made by Holman Devin et al. [22], who observed a decrease of ARGs of various antimicrobial classes post-weaning in an age-dependent manner. Resistance to MLS_B_ (macrolides, lincosamides and streptogramin B) and tetracycline were reported as more stable in their study due to the broader host-range of these ARGs. These changes in AMR richness and diversity were also observed in the beta diversity analysis of in the present study.

While the results of the two sample points post-weaning of AB-ZnO free farms closely clustered together in the NMDS plot, sample points from AB-ZnO farms were much more dispersed. These results were however not significant according to the pairwise PERMANOVA analysis. Instead, significant differences between resistomes were mainly observed between environmental and faecal samples, which could be associated with differing microbiomes and the fact that cleaned pen were sampled before piglets entered the pens [20]. Nevertheless, it is assumed that changes in the microbiome post-weaning additionally affect the resistome [22]. Variation in the resistome could be associated with the changing microbiomes in the absence of milk as the pigs adapt to solid feed until the microbiome stabilises, resulting in reduced variation of the resistome [28–30]. However, this natural adaption process may be impacted by AMU and ZnO usage, resulting in a higher variation of the resistome on AB-ZnO farms compared to AB-ZnO free farms [29]. Furthermore, the AB-ZnO farms used different antimicrobials in combination with ZnO, which may have resulted in a higher variation between samples due to differing selective pressures.

In the second part of the study, contigs were divided into chromosomal and plasmidic contigs using the software Platon. This was based on the hypothesis that significant differences between treatment types may become indistinct or less visible due to associations of ARGs with either the microbiome (chromosomal encoded ARGs) or MGEs [22]. Further comparison of sample and treatment types in the form of heatmaps and clustering based on Euclidean distance resulted in a clear separation of environmental samples and faecal samples for both chromosomal and plasmidic contigs, similar to the beta diversity analysis. Interestingly, chromosomal BRGs and MRGs were observed in greater amounts in faecal samples. This might be due to a bias in the BacMet2 database of experimentally confirmed genes towards model microorganisms such as *E. coli* [31]. However, a high abundance of BRGs and MRGs has been reported before in 2-day-old piglets in a study by Gaire et al. [32]. The reasons for this remain unknown, but it may provide an explanation for their occurrence in young piglets.

Subsequent analysis of integrons showed that class 1 and class 2 integrons were the main carriers of ARGs and BRGs. Similar to the observation of Mencía-Ares et al. [20], the ARGs *aadA* and *dfrA* were frequently observed on integrons. In addition, the BRGs *qacF* and *qacEΔ1,* and the ARGs *cmlA* and *ant(3'')-Ia* were found on integrons. These genes have been previously reported in pig farm isolates [33–35]. Moreover, no difference in the prevalence of ARG-carrying integrons was observed between treatment types. Similar to our findings, Mencía-Ares, et al. [20] rarely observed integrons in faecal samples. It is unclear why in the current study the majority of integrons was found in environmental samples, while the majority of BRGs was detected in faecal samples. BRGs and integrons may be carried by differing taxa or be affected by co-selection [32, 36]. Thus, future studies should further investigate the presence and absence of BRGs and integrons in pigs.

In the last part of this study, MAGs were generated to examine associations of resistance genes with taxa at species level to identify potential carriers of resistance genes on pig farms. The first observations that were made based on ARG prevalence, when comparing AB-ZnO farms with AB-ZnO free farms, was that the latter had an overall greater diversity of species and ARGs. AB-ZnO farms had a greater diversity of Actinomycetota species, or more specifically *Corynebacterium* spp. These observations have been described before in two studies by Yu et al. [37] and Jo et al. [38], which used MLSP antimicrobials in the form of an in-feed antimicrobial cocktail (50 mg/kg olaquindox, 50 mg/kg oxytetracycline calcium, and 50 mg/kg kitasamycin) or in feed lincomycin alone (lincomycin 0.1%, 1 kg/ton, sub-clinical), respectively. Thus, ZnO and sulphadiazine-trimethoprim may co-select for MLSP resistant species, resulting in an increase of *Corynebacterium* species [9, 23]. Interestingly, a greater diversity of beta-lactam resistance genes was observed on AB-ZnO free farms compared to AB-ZnO farms despite the fact that three AB-ZnO farms used amoxicillin as a prophylactic treatment. Although it is unclear if synergistic effects of ZnO in combination with amoxicillin exist, it is reasonable to suggest that the combination of both co-selects for species which show resistance to both and thereby may alter the genetic pool of beta-lactam resistance mechanisms. However, it is difficult to base these assumptions on a limited number of MAGs. Further investigation will be needed in the future.

Furthermore, MRGs and BRGs were much more diverse on AB-ZnO farms, suggesting that ZnO and/or antimicrobial usage may promote the spread and acquisition of MRGs and BRGs. However, it should be considered that most of the MRGs and BRGs were associated with *E. hormaechei*, an *Enterobacteriaceae* species, which may, similar to *E. flexneri,* cause a significant bias in the observations of this study related to the BacMet2 database. Nevertheless, our study was able to associate ARGs, BRGs, and MRGs with specific taxa, of which some are able to colonise humans and animals, and have the potential to cause disease, and thereby pose a health risk for animals, farmers, and consumers.

Summarizing, few significant differences between treatment types were observed in this study although a few associations between antimicrobial and ZnO usage with AMR prevalence were observed. It could be that due to the limited sample size and differing treatments on AB-ZnO farms significant differences could not be observed. Moreover, the combination of antimicrobial and ZnO treatment on AB-ZnO farms may have further affected the results, as it is unclear to date if ZnO has any synergistic or inhibitory interactions with antimicrobials, which were used in this study. Furthermore, considering that only combined treatments were administered, it remains elusive if ZnO or antimicrobials had a greater effect on the resistome and how it was affected by each individual component. Apart from these few differences detected, sample type, and age of piglets were the predominant influences on AMR prevalence as previously reported by by Holman Devin et al. [22], which could be primarily due to correlations between the microbiome and the resistome. Considering these correlations, AMU and ZnO usage may cause disruptions in the microbiome, resulting in higher variations 2-weeks post-weaning compared to non-treated piglets. Moreover, considering the previous AMU on AB-ZnO free farms prior to the 3-year free period, limited evidence was seen that removal of AMU and ZnO from regular farm practices reduced AMR on farms within 3 years. It is possible that more time will be needed after removal of antimicrobials and ZnO to see clear differences in AMR prevalence. Additionally, regular farm practices such as the use of biocides could co-select for ARGs, thereby potentially delaying the desired reduction of AMR, or may promote its persistence. In addition, our study used a limited number of samples which could explain the limited number of statistically significant observations made.

## Conclusion

In this study, few significant differences between commercial AB-ZnO free and AB-ZnO farms in terms of AMR prevalence were observed in accordance with previous studies. Based on contig- and MAG-based observations, the microbiome and MGEs such as plasmids and integrons, both of which may be affected by AMU and ZnO use, appeared to be the main contributors to these significant differences. The age of piglets and sample type played a crucial role regarding the composition of the resistomes on farms. Future studies may consider increasing sample numbers and focus on the effects of more long-term removal of AMU and ZnO from on-farm protocols to further investigate its effectiveness in the fight against AMR.

## Methodology

### Experimental design and sampling procedure

In this observational longitudinal study, environmental and faecal samples were taken from ten commercial farms in the Republic of Ireland. Five of these farms used ZnO (3000 ppm) and antimicrobials, namely sulphadiazine-trimethoprim (Sulfoprim 15% [15 mg/kg], Univet Limited, Ireland; on Farm1 and Farm3) or amoxicillin (Stabox [15 mg/kg], Virbac, France; on Farm4, Farm6, and Farm8) as a 2-week post-weaning treatment, the other five farms did not use any antimicrobial or heavy metal post-weaning for the last 3 years. Samples were taken on each farm from two physically separated pens in different rooms. Samples collected per farm included: (i) two faecal samples at the day of weaning (FW0), (ii) two faecal samples 1 week post-weaning (FW1), (iii) two faecal samples 2 weeks post-weaning (FW2), (iv) one swab samples of cleaned feeders and drinkers (SFD) and (v) one walls and floor of cleaned pens before transfer of pigs (SWF). Thus, on each farm six faecal samples and two environmental samples were taken. For all time points on each farm, one faecal sample was randomly collected from two different rooms immediately after excretion and transferred into 1.5 mL Eppendorf tubes for later processing and pooling. Swab samples were collected by using sponge-sticks soaked in 10 mL neutralizing buffer (3M™, Ireland). For SFD samples no defined area was swabbed, while for the SWF samples an area of approximately 50 cm^2^ was swabbed. All samples were cooled for transportation and processed within 24 h. Swab samples were mixed with 5 mL sterile Phosphate Saline Buffer 1X (1 × PBS) prior to extracting 8 mL and 16 mL of the SWW and SWD swabs respectively. Similarly, faecal samples in Eppendorf tubes were centrifuged at 3000×g and 4 °C for 15 min. After discarding the supernatant, pellets were resuspended in 1 mL 1 × PBS and stored together with swab samples at − 80 °C.

### DNA extraction, library preparation, and sequencing

Prior to DNA extraction, environmental samples were centrifuged at 15.000 rpm at 4 °C for 1 min and the supernatant discarded. For DNA extraction from faecal and environmental swab samples, either 200 ± 50 mg of faecal matter or the obtained pellet from the swab samples was used with the QIAamp PowerFecal Pro DNA Kit (Qiagen, Crawley, West Sussex, UK). DNA extracts were quantified using a Qubit 3.0 Fluorometer (Invitrogen, ThermoFisher Scientific, United Kingdom). Samples per farm were pooled together according to sample type by using an aliquot of 5 µL of 1 ng/µL DNA for each pooled sample. The Illumina Nextera XT Library Preparation Kit (Illumina Inc., San Diego, CA) was used according to instructions of the manufacturer to construct paired-end sequencing libraries and libraries were assessed using an Agilent Technology 21000 Bioanalyzer. Libraries were pooled equimolarly and sequenced in Teagasc Food Research Centre Moorepark on the Illumina NextSeq 500 platform (2 × 150 bp, v2). Samples had a sequencing depth of 5.2–9.4 million reads (average: 7.7 million reads; excluding negative controls).

### Bioinformatics analysis

The quality of raw read sequences was evaluated with FastQC (v0.11.8) and MultiQC (v 1.9) [39, 40]. Subsequently, sequence contamination including host and PhiX phage DNA were removed using Bowtie2 (v2.4.4) [41], SAMtools (v1.9) [42], and BEDtools (v2.27.1) [43] together with the reference sequences for *Sus scrofa* (Sscrofa11.1 assembly; RefSeq accession number: GCF_000000215.6) and the PhiX phage (NC_001422). Unmapped reads were further processed and quality trimmed using Trimmomatic (v0.38) [44] to obtain only reads with a minimum Phred score of 33 and a minimum length of 80 bp. Nextera adapter sequences were additionally removed with Trimmomatic. Trimmed sequences were assembled to contigs using metaSPAdes (v3.15.3) [45]. Short contigs below 1000 bp were removed using BBMap (v38.22) and the reformat.sh command (minlength = 1000) [46].

Contigs were then examined for ARGs using ABRicate (https://github.com/tseemann/ABRicate; v1.0.1) together with the ResFinder database (last update: 2021-Mar-27) using a minimum identity of 80% and a minimum coverage of 75%. The presence of MRGs and BRGs were confirmed using Bacmet-Scan (v1.0) with default settings and the BacMet2 database containing experimentally confirmed resistance genes [31]. Resistance classes were specified using the manually curated ResFinder repository published by Mencía-Ares et al. [20] and the classifications provided by the Bacmet2 database website (http://bacmet.biomedicine.gu.se/). For four genes, namely *mdf*(A)*, mdt*(A)*, oqxA,* and *oqxB*, hits via the ResFinder and BacMet2 database were obtained. Due to differing results based on screening sensitivity, only hits for these genes using the ResFinder database were included in the analysis. Contigs were screened for plasmids, integrons, and horizontal gene transfer events using Platon (v1.6) [18], IntegronFinder (v2.0) [47], and WAAFLE (v1.0; http://huttenhower.sph.harvard.edu/waafle) respectively.

Resistance genes on chromosomal, plasmidic, and integron sequences, as well as on transferred elements were confirmed using ABRicate, BacMet2, and the mobilome analysis pipeline created by José F. Cobo-Díaz (https://github.com/JoseCoboDiaz/ARG-contig_mobilome_analysis). MAGs were assembled by back-mapping trimmed reads to contigs using Bowtie2 and binning of mapped reads with a minimum length of 1500 bp using MetaBAT2 (v2.12.1) [48]. Quality assessment of bins was performed using CheckM (v1.0.18) [49] and bins with a completeness < 50% and contamination > 5% were excluded from the further analysis. Taxonomic classification of MAGs was performed using GTDB-Tk (v1.5.0) [50] and again ABRicate and BacMet2 were used with the same settings as described before to identify resistance genes on MAGs.

### Visualization and statistical methods

Statistical analysis and data visualization were performed using R (v4.0.2) and RStudio (v2021.09.2) [51]. The majority of plots were visualized using the ggplot2 package [52]. Statistical comparison between different samples of AB-ZnO free and AB-ZnO farms were performed using the Mann–Whitney U test (R package: rstatix v0.7.0) [53]. Dissimilarity analysis based on Bray–Curtis dissimilarities and non-metric multidimensional scaling (NMDS) were performed using the R vegan package (v2.6-2) [54]. Statistical comparison between group dissimilarities was conducted using the ‘pairwise.adonis2’ function of the R pairwiseAdonis package (v0.4.1) [55] for pairwise PERMANOVA analysis. Gene maps of integrons harbouring more than one resistance gene were created using the R gggenes package (v0.4.1) [56] and heatmaps were generated using the R pheatmap package (1.0.12) respectively [57]. Chord diagrams of MAGs were created using the R circlize package (v0.4.14) [58]. All graphs were arranged to figures and improved in quality by using the R ggpubr package (v0.4.0) [59] and Affinity Designer (v1.8.5.703; Serif).

### Supplementary Information

**Additional file 1: **List of total MAGs and associated antimicrobial, heavy metal, and biocide resistance genes. The excel tables include sample IDs, binned MAGs per sample, taxonomic assignment, and found resistance genes on binned MAGs.

## Data Availability

Raw sequences of the metagenomics analysis are publicly accessible under BioProject: PRJNA894343.
